# Super-enhancer profiling identifies novel critical and targetable cancer survival gene LYL1 in pediatric acute myeloid leukemia

**DOI:** 10.1186/s13046-022-02428-9

**Published:** 2022-07-16

**Authors:** Fang Fang, Jun Lu, Xu Sang, Yan-Fang Tao, Jian-Wei Wang, Zi-Mu Zhang, Yong-Ping Zhang, Xiao-Lu Li, Yi Xie, Shui-Yan Wu, Xin-Ran Chu, Gen Li, Di Wu, Yan-Ling Chen, Juan-Juan Yu, Si-qi Jia, Chen-xi Feng, Yuan-Yuan Tian, Zhi-Heng Li, Jing Ling, Shao-Yan Hu, Jian Pan

**Affiliations:** 1 Institute of Pediatric Research, Children’s Hospital of Soochow University, No.92 Zhongnan Street, SIP, Suzhou, 215003 China; 2 Department of Hematology, Children’s Hospital of Soochow University, No.92 Zhongnan Street, SIP, Suzhou, 215003 Jiangsu China; 3 Department of Pediatrics, The First Affiliated Hospital of Bengbu Medical College, Bengbu, 233004 China; 4 Intensive Care Unit, Children’s Hospital of Soochow University, Suzhou, 215003 China

**Keywords:** Acute myeloid leukemia, Super-enhancer, LYL1, GNE-987, ChIP-Seq analysis

## Abstract

**Background:**

Acute myeloid leukemia (AML) is a myeloid neoplasm makes up 7.6% of hematopoietic malignancies. Super-enhancers (SEs) represent a special group of enhancers, which have been reported in multiple cell types. In this study, we explored super-enhancer profiling through ChIP-Seq analysis of AML samples and AML cell lines, followed by functional analysis.

**Methods:**

ChIP-seq analysis for H3K27ac was performed in 11 AML samples, 7 T-ALL samples, 8 B-ALL samples, and in NB4 cell line. Genes and pathways affected by GNE-987 treatment were identified by gene expression analysis using RNA-seq. One of the genes associated with super-enhancer and affected by GNE-987 treatment was LYL1 basic helix-loop-helix family member (*LYL1*). shRNA mediated gene interference was used to down-regulate the expression of *LYL1* in AML cell lines, and knockdown efficiency was detected by RT-qPCR and western blotting. The effect of knockdown on the growth of AML cell lines was evaluated by CCK-8. Western blotting was used to detect PARP cleavage, and flow cytometry were used to determine the effect of knockdown on apoptosis of AML cells.

**Results:**

We identified a total of 200 genes which were commonly associated with super-enhancers in ≧10 AML samples, and were found enriched in regulation of transcription. Using the BRD4 inhibitor GNE-987, we assessed the dependence of AML cells on transcriptional activation for growth and found GNE-987 treatment predominantly inhibits cell growth in AML cells. Moreover, 20 candidate genes were selected by super-enhancer profile and gene expression profile and among which *LYL1* was observed to promote cell growth and survival in human AML cells.

**Conclusions:**

In summary, we identified 200 common super-enhancer-associated genes in AML samples, and a series of those genes are cancer genes. We also found GNE-987 treatment downregulates the expression of super-enhancer-associated genes in AML cells, including the expression of *LYL1*. Further functional analysis indicated that *LYL1* is required for AML cell growth and survival. These findings promote understanding of AML pathophysiology and elucidated an important role of *LYL1* in AML progression.

**Supplementary Information:**

The online version contains supplementary material available at 10.1186/s13046-022-02428-9.

## Background

Acute myeloid leukemia (AML) is a myeloid neoplasm that accounts for 7.6% of hematopoietic malignancies. In bone marrow (BM), AML arise from oncogenic transformation of hematopoietic progenitors which damages the blood tissue. According to reports, the long-term survival rate of patients with AML is less than 20% [1–3]. It has also been reported that approximately18,000 AML cases are diagnosed each year in Europe [4]. The roles of multiple genes are different between pediatric AML and adult AML. AML is complex, and exploring its pathogenic mechanisms will help improve the current state of AML treatment [5–8].

A series of hub genes have been identified in AML. RUNX family transcription factor 1 (RUNX1) dysfunction is reported to be one of the major pathogenic mechanisms of AML [9]. RUNX1 point mutations have been identified in myelodysplastic syndrome-related AML. It has been reported that somatic RUNX1 mutations have been found in approximately 10% of patients with de novo AML [10]. Myeloperoxidase (MPO) has been widely accepted as a marker for AML diagnosis, and it is also associated with AML prognosis [11]. Cyclin-dependent kinase 6 (CDK6) is another key molecule in the development of AML. It functions as a driver of mixed-lineage leukemia rearrangements [12].

Pediatric AML is different from adult AML as their biological process and clinical prognoses are distinct [13, 14]. Childhood AML is reported to have fewer somatic mutations and more cytogenetic abnormalities than adult AML. The epigenetic landscapes of pediatric and adult AML are also different. Furthermore, differences in prognosis between childhood AML and adult AML have also been reported.

Super-enhancers (SEs) represent a special group of enhancers that have been reported in multiple cell types [15]. Super-enhancers recruit a particularly large number of transcription factors/cofactors and induce the transcription of many target genes, compared with typical enhancers (TEs). H3k27ac is one of the frequently-used indicators for super-enhancer identification [15]. Aberrant expression of genes triggered by super-enhancers participates in many biological processes, therefore the screening and identification of hub genes driven by super-enhancers attracted the attention of many researchers.

Super-enhancers have been reported to be implicated in multiple types of cancers. Super-enhancer promotes the growth and survival of t(4;14)-positive multiple myeloma [16]. HJURP was reported to be an SE-associated gene in t(4;14)-positive multiple myeloma. Super-enhancer activates the histone chaperone HJURP, which leads to abnormal overexpression of HJURP in t(4;14)-positive multiple myeloma. Overexpression of HJURP further promotes tumor cell proliferation and is associated with poor outcome in t(4;14)-positive multiple myeloma. Super-enhancer was found to activate the Wnt/beta-catenin pathway and promotes the proliferation of liver cancer cells [17]. In hepatocellular carcinoma specimens, a live-specific super-enhancer drives lncRNA-DAW, leading to activation of the Wnt/beta-catenin pathway. Oncogenic super-enhancers were also identified in colorectal cancer through genome-wide profiling [18]. Via a genome-wide investigation of the enhancer distribution in colorectal cancer tissues, super-enhancer loci were identified. Super-enhancers were found to govern PHF19 and TBC1D16 and participate in colorectal cancer tumorigenesis. In addition, super-enhancer was reported to play a role in glioma progression [19]. In glioma cells, TMEM44-AS1 activates Myc signaling, and Myc binds to the super-enhancer of TMEM44-AS1, forming a positive feedback loop. Myc was reported to interact with mediator complex subunit 1 and regulate the super-enhancer of TMEM44-AS1 in glioma cells. The small molecule Myc inhibitor, Myci975, can alleviate glioma cell growth promoted by TMEM44-AS1. Super-enhancer was also found to be involved in squamous cell carcinoma [20]. Super-enhancers were reported to form at cancer stemness genes and disruption of super-enhancers using BET inhibitors was reported to inhibit the self-renewal of cancer stem cells in head and neck squamous cell carcinoma. Super-enhancer was reported to control the expression of TP63, which is involved in cancer stem cell self-renewal in head and neck squamous cell carcinoma. BRD4 was reported to recruit MED1 and p65 to form super-enhancers, and a BRD4 inhibitor was reported to disrupt super-enhancers and decrease the tumorigenic potential of cancer stem cells in head and neck squamous cell carcinoma. Furthermore, super-enhancer is known to play a role in triple-negative breast cancer [21]. The super-enhancer heterogeneity in breast cancer subtypes was uncovered through multiomic profiling. Certain genes (including FOXC1, MET, and ANLN) were identified to be regulated specifically by triple-negative breast cancer-specific super-enhancers. A super-enhancer-driven master regulator of invasion and metastasis was identified in triple-negative breast cancer. In addition, super-enhancer is reported to be abnormally activated and result in CHPT1 overexpression, which leads to enzalutamide resistance in castration-resistant prostate cancer [22].

In this study, we performed super-enhancer profiling through ChIP-Seq analysis of AML cell lines and AML samples, followed by functional analysis. We identified 200 common super-enhancer-associated genes in AML samples, and a series of those genes are cancer genes. We also found that GNE-987 treatment downregulates the expression of super-enhancer-associated genes in AML cells, including the expression of *LYL1*. Further functional analysis indicated that *LYL1* is required for AML cell growth and survival. These findings provide novel insights into the pathophysiology of AML and elucidate a crucial role of *LYL1* in promoting AML progression.

## Materials and methods

### Samples

This study was performed according to The Code of Ethics of the World Medical Association (Declaration of Helsinki). The ethics committee of Children’s Hospital of Soochow University approved this study (No.SUEC2000–021 & No.SUEC2011–037). Written informed consent was obtained from each participating individual’s guardian. A total of 11 pediatric AML bone marrow samples, 7 T-ALL bone marrow samples, and 8 B-ALL bone marrow samples were collected, and H3K27ac signals were detected by ChIP-Seq. The clinical characteristics of the patients are shown in Table 1 and Supplementary Table 1.

**Table 1:** Clinical characteristics of pediatric patients included in ChIP-seq analysis

	Pediatric patients
*AML patients*	
Age (year)	8.92 ± 3.12
Sex(Male/Female)	4/7
WBC (10^9/L)	23.00(6.35–59.60)
*T-ALL patients*	
Age (year)	7.7 ± 3.83
Sex(Male/Female)	6/1
WBC (10^9/L)	227.77 (30.30–616.63)
*B-ALL patients*	
Age (year)	5.47 ± 3.40
Sex(Male/Female)	5/3
WBC (10^9/L)	45.85 (10.10–137.00)

### Cell lines and culture

Human AML cell lines, including NB4, Kasumi-1, MV4–11, and HL-60 were acquired from the cell bank of Chinese Academy of Sciences. Cells were cultured at 37 °C in RPMI medium (Thermo Fisher Scientific) supplemented with 1% penicillin–streptomycin (Beyotime Biotechnology, Shanghai, China) and 10% fetal bovine serum (Biological Industries, CT, USA), in a humidified incubator with 5% CO_2_.

### Cell viability assay

Leukemia cells were seeded in the 96-well plate and GNE-987 was added at different concentrations. Control group cells were treated with 0.05% dimethyl sulfoxide (DMSO) without GNE-987 in complete medium. Cell viability was determined by a Cell Counting kit-8 (CCK8) assay (Dojindo Molecular Technologies, Tokyo, Japan) according to the manufacturer’s instructions after 24 h of drug treatment. Each concentration was tested in three independent experiments. Cell proliferation was quantified using Graph Prism 7.0 (GraphPad Software Inc., San Diego, CA, USA).

### Lentivirus preparation and infection

We constructed short hairpin RNA (shRNA) targeting *LYL1* (shown in Supplementary Table 2) using the pLKO.1-puro lentiviral vector (IGE Biotechnology Ltd., Guangzhou, China). We also constructed PLVX-LYL1 (shown in Supplementary Table 2) in this study. To prepare lentivirus, we purchased the envelope plasmid and packaging plasmid from Addgene (pMD2.G: #12,259; psPAX2:#12,260; Cambridge, MA, USA). Next we cotransfected pMD2.G, psPAX2 and the transfer plasmid into 293FT cells by polyethylenimine (linear MW 25,000 Da, 5 mg/mL, pH 7.0) (cat. No. 23966–1; Polysciences, Warrington, PA, USA). Then we replaced the entire volume of culture medium with fresh medium after 6 h. Next we harvested the viral supernatant at 48 h post-transfection and filtered it through a 0.22 μm filter. Then we infected the leukemia cells with lentivirus in the presence of 10 μg/mL Polybrene (Sigma-Aldrich) for 24 h. Next, we selected stable cells using puromycin (Sigma-Aldrich).

### RNA preparation and real-time PCR

We extracted total RNA with TRIzol® reagent (Invitrogen, CA, USA). Next we reverse transcribed total RNA to cDNA with a High-Capacity cDNA Reverse Transcription Kit (Applied Biosystems, CA, USA). Quantitative real-time PCR was performed with LightCycler® 480 SYBR Green I Master mix (cat. No. 04707516001; Roche, Penzberg, Germany) in a LightCycler 480 Real Time System (Roche). Then, we calculated mRNA expression levels according to the Ct method, using glyceraldehyde 3-phosphate dehydrogenase (GAPDH) expression as the internal reference. The primer sequences used in this study are listed in Supplementary Table 3.

### Western blotting analysis

Western blotting analysis was performed with primary antibodies specific for: BRD2 (cat. No. 5848 s; 1:1000; Cell Signaling Technology, Boston, MA, USA), BRD3 (cat. No. 11859–1-AP; 1:1000; Proteintech, Chicago, IL, USA), BRD4 (cat. No. 13440 s; 1:1000; Cell Signaling Technology), LYL1((C-4):sc-374,164; Santa Cruz Biotechnology), and PARP (cat. No. 9542; 1:1000; Cell Signaling Technology), with an antibody specific for glyceraldehyde 3-phosphate dehydrogenase (GAPDH) (cat. No. MA3374; 1:1000; Millipore) as a reference protein. Peroxidase-conjugated Afniure goat anti-rabbit IgG (H + L) (cat.111–035-003; 1:5000) and goat anti-mouse IgG (H + L) (cat. No. 115–035-003; 1:5000) secondary antibodies were purchased from Jackson ImmunoResearch Laboratories, Inc. (West Grove, PA, USA).

### Apoptosis assay

We harvested leukemia cells and washed them with cold PBS. Then, the cells were suspended in 1 × binding buffer, and stained with fuorescein isothiocyanate (FITC)-Annexin V antibody and PI solution by the FITC-Annexin V apoptosis kit (cat. No.556420; BD Biosciences, Franklin Lakes, NJ, USA). Apoptosis was analyzed using flow cytometry (Beckman Gallios™ Flow Cytometer; Beckman).

### RNA-seq analysis and data processing

RNA-seq was performed using protocols from Novogene Bioinformatics Technology Co., Ltd. (Beijing, China). Library construction was the first step, with reverse transcription of total RNA to cDNA. Next, the cDNA library was sequenced. We then filtered the raw reads and mapped the clean reads using HISAT. Next we calculated gene expression level and identified differentially expressed genes with DESeq2 (*P* < 0.05 and fold-change> 2 or fold-change< 0.5). Differentially expressed genes were further analyzed with the R package clusterProfiler [23] and the DAVID Bioinformatics Resources v6.8 online server (https://david.ncifcrf.gov) for enrichment analysis.

### Chromatin immunoprecipitation (ChIP)

We crosslinked 3–5× 10^7^ cells with 1% formaldehyde for 10 minutes, and then quenched the crosslinking reaction with 1.25 M glycine at room temperature for 5 minutes. Fixed cells were then harvested, lysed, and sonicated with the Bioruptor (Diagenode, Liège, Belgium). Sonicated chromatin was next incubated with an anti-histone H3 (acetyl K27) antibody (cat. No. ab4729; Abcam, Cambridge, UK) overnight at 4 °C. DNA was eluted and purified with a QIAquick PCR purification kit (cat. No. 208106; Qiagen, Hilden, Germany). Samples were sequenced using the BGISEQ 2000 platform (Beijing Genomics Institute (Shenzhen, China)) and the NovaSeq 6000 platform (Novogene Bioinformatics Technology Co., Ltd. Beijing, China). We aligned the raw ChIP-Seq H3K27ac data to UCSC hg38 (the reference genome) with Bowtie2 (v 2.3.5) [24], according to the alignment parameters -p 4 -q -x. Peaks were then identified with MACS2 (v2.0.9) [25], according to the parameters -g hs -n test -B -q 0.01. Next we converted the bedgraph files generated by MACS2 to bigwig files with the UCSC bedGraphToBigWig tool, and then visualized the bigwig files using Integrative Genomics Viewer (IGV) [26]. Then we identified super-enhancers by the ROSE (Rank Order of Super Enhancers) method [27, 28], according to the parameters -s 12500 -t 2000 (−s, stitching distance; −t, TSS exclusion zone size).

### Public ChIP-Seq data collection and analysis

In the present study, the Cistrome database (http://www.cistrome.org/) was used to search public ChIP-Seq H3K27ac datasets for AML cell lines. The Cistrome database was also used to search LYL1 ChIP-Seq datasets in AML cell lines. Raw data from these public ChIP-Seq datasets were downloaded and aligned to UCSC hg38 (the reference genome) with Bowtie2 (v 2.3.5) [24], according to the parameters -p 4 -q -x. Peaks were obtained with MACS2 (v2.0.9) [25], according to the parameters -g hs -n test -B -q 0.01.The bigwig files of these datasets (GSE80779, GSE123872, GSE63484) [29–31] were next visualized using Integrative Genomics Viewer (IGV) [26].

### Public hi-C data collection and analysis

Hi-C data for THP-1 cell line (GSE126979) were downloaded from the Gene Expression Omnibus database. Read mapping and loop calling were performed with HiC-pro (v.3.1.0) [32]. For alignment, MboI restriction sites in the hg38 build were used. HiC-pro uses Bowtie2 for mapping and we specified –very-sensitive -L 30 --score-min L,-0.6,-0.2 --end-to-end –reorder for global options and --very-sensitive -L 20 --score-min L,-0.6,-0.2 --end-to-end –reorder for local options. We used ‘GATCGATC’ as the ligation site during the mapping process. The results of this analysis were visualized and graphed using WashU tool (http://epigenomegateway.wustl.edu/browser/).

### Statistical analysis

Student’s t-test or the Mann-Whitney u test was used for comparisons between two groups. Statistically significant *P* values are labeled as follows: * for *P* < 0.05, ** for *P* < 0.01, and *** for *P* < 0.001. Statistical analysis was performed using GraphPad Prism 7.0 (GraphPad Software, Inc., La Jolla, CA, USA).

## Results

### Super-enhancers are enriched at transcriptional regulatory genes in AML samples and AML cells

**Fig. 1:**
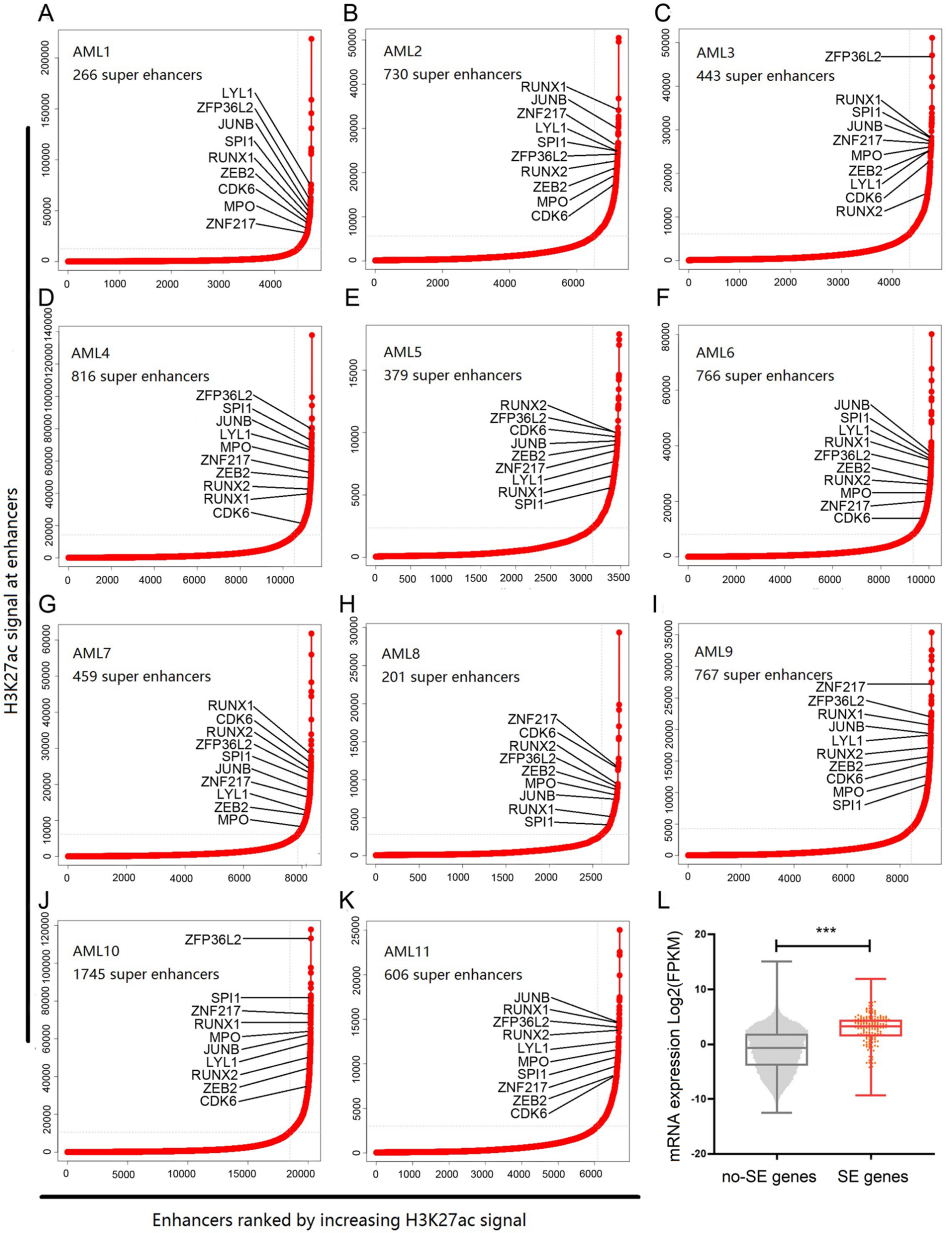
**A-K** Super-enhancer profiling in AML samples. Enhancers were ranked by increasing H3K27ac signal in 11 AML samples. Number of super-enhancers identified in each AML sample is shown. Examples of genes commonly associated with super-enhancers in ≧10 AML samples are shown. **L** RNAseq results of 9 AML samples suggested that the expression levels of the 200 super-enhancer-associated genes were significantly higher than other genes

**Fig. 2:**
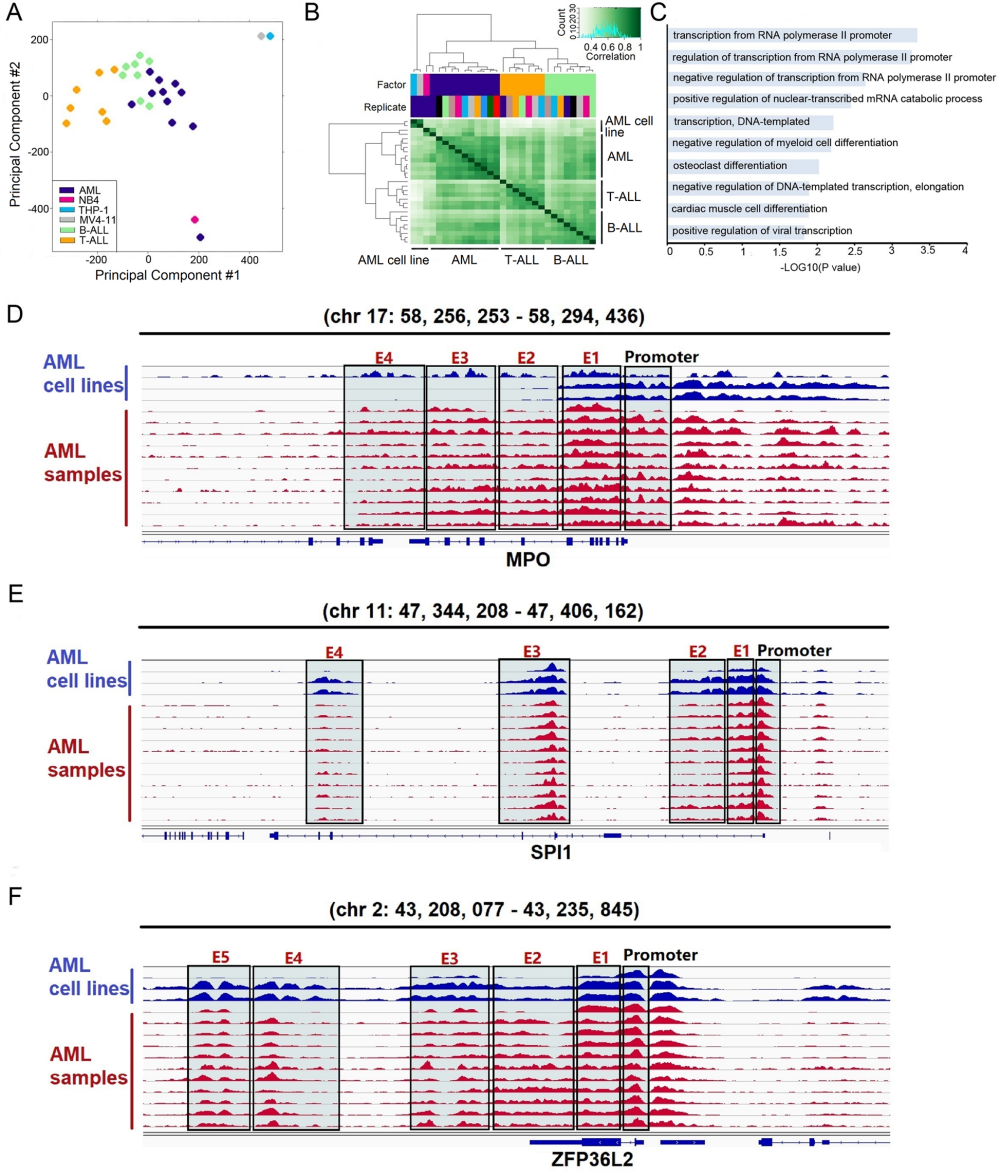
**A** PCA was performed for 11 AML samples, 7 T-ALL samples, 8 B-ALL samples, and 3 AML cell lines based on the H3K27ac signals identified in each sample. Each circle represents a sample, and each color represents the type of sample. **B** Cluster analysis results of 11 AML samples, 7 T-ALL samples, 8 B-ALL samples, and 3 AML cell lines based on the H3K27ac signals identified in each sample. **C** genes commonly associated with super-enhancers in AML samples were subjected to gene ontology analysis. The significant categories are shown. **D** the ChIP-Seq gene tracks represent the H3K27ac signal in AML cell lines (NB4, MV4–11, and THP1) and 11 AML samples at the *MPO* gene loci. The super-enhancers are shown as green boxes. **E** the ChIP-Seq gene tracks represent the H3K27ac signal in AML cell lines (NB4, MV4–11, and THP1) and 11 AML samples at the *SPI1* gene loci. The super-enhancers are shown as green boxes. **F** the ChIP-Seq gene tracks represent the H3K27ac signal in AML cell lines (NB4, MV4–11, and THP1) and 11 AML samples at the *ZFP36l2* gene loci. The super-enhancers are shown as green boxes

To identify genes correlated with super-enhancers in AML, we carried out H3K27ac ChIP-seq analysis in 11 AML samples (Table 1 and Supplementary Table 1). In this study NB4, MV4–11 and THP-1 cells were also used as representative AML cell lines. For these 3 AML cell lines, we carried out H3K27ac ChIP-seq analysis in NB4 cell line, and also analyzed public H3K27ac ChIP-seq datasets for MV4–11 and THP-1 cell lines (GSE80779 and GSE123872) [29, 30]. Additionally, we included 7 T-ALL samples and 8 B-ALL samples, to compare the H3K27ac signals with those in AML samples. Putative super-enhancers identified in each of the 11 AML samples are shown in Fig. 1A-K and Supplementary Table 4, with RNAseq results for 9 of the 11 AML samples shown in Fig. 1L. Putative super-enhancers identified in the 7 T-ALL samples and 8 B-ALL samples are shown in Supplementary Tables 5 and 6. The principal component analysis (PCA) result and clustering result based on the peak signals clearly distinguished AML samples from T-ALL or B-ALL samples (Fig. 2A and B). Next, a total of 200 genes were selected which were commonly correlated with super-enhancers in ≧10 AML samples (Supplementary Table 7). Gene ontology enrichment analysis suggested that these 200 genes were enriched in regulation of transcription and regulation of myeloid cell differentiation (Fig. 2C, Supplementary Table 8). RNAseq results of 9 AML samples suggested that the expression levels of the 200 super-enhancer-associated genes were significantly higher than those of the other genes (Fig. 1L, Supplementary Table 9). Our results indicate that the genes commonly correlated with super-enhancers in AML are generally involved in the regulation of transcription.

### Super-enhancers in AML are associated with the known cancer genes

Among the 200 above-mentioned genes, a series of genes have been determined to be involved in cancers. For instance, super-enhancers were present at the *MPO* gene locus in 10 AML samples and 3 AML cell lines (NB4, MV4–11, and THP-1) (Fig. 2D). Similarly, we found super-enhancers at the *SPI1* locus in 11 AML samples and 3 AML cell lines (NB4, MV4–11, and THP-1) (Fig. 2E). Additionally, super-enhancers were observed at the *ZFP36L2* gene locus in all AML samples (Fig. 2F). Compared to T-ALL or B-ALL, super-enhancers associated with *MPO* were observed to be AML specific, while super-enhancers associated with *ZFP36L2* were common to all three hematological diseases (AML, T-ALL, and B-ALL) (Supplementary Figs. 1 and 2). In addition, compared to T-ALL, super-enhancers associated with *SPI1* were found to be AML and B-ALL specific (Supplementary Fig. 3). According to these findings, these gene loci are particularly activated in AML. Although the sample size of this cohort was small, our results indicate that super-enhancers are correlated with cancer genes and lead to abnormal activation of those genes during AML progression.

### The BRD4 inhibitor GNE-987 inhibits AML cell growth

**Fig. 3:**
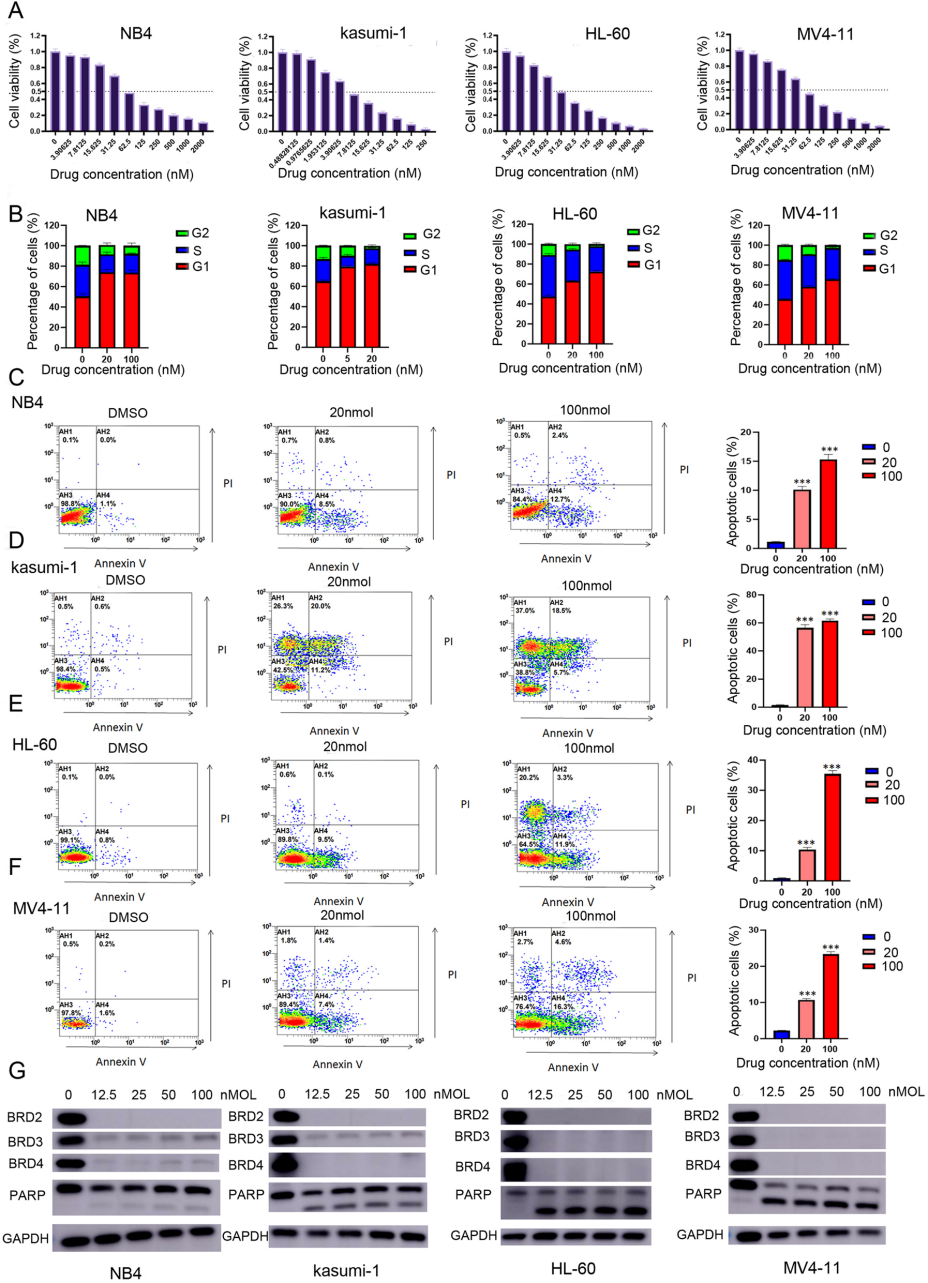
GNE-987 restained the proliferation of AML cell lines. **A** drug sensitivity assay of NB4, Kasumi-1, HL-60, and MV4–11 cell lines after treatment with gradient concentrations of GEN987 for 24 h. **B** GNE-987 blocked cell cycle of AML cells. Cell cycle of NB4, Kasumi-1, HL-60, and MV4–11 cell lines were analyzed after treatment with DMSO or GNE-987 for 24 h. AML cells were distributed in G1/S phase and the cell population in G1 phase increased dramatically after treatment with GNE-987. **C** Annexin V and PI-labeled cell apoptosis of NB4 cell line analyzed by flow cytometry after DMSO or GNE-987 treatment for 24 h. The apoptotic rates of NB4 cell line were significantly increased after GNE-987 treatment. **D** Annexin V and PI-labeled cell apoptosis of Kasumi-1 cell line analyzed by flow cytometry after DMSO or GNE-987 treatment for 24 h. The apoptotic rates of Kasumi-1 cell line were significantly increased after GNE-987 treatment. **E** Annexin V and PI-labeled cell apoptosis of HL-60 cell line analyzed by flow cytometry after DMSO or GNE-987 treatment for 24 h. The apoptotic rates of HL-60 cell line were significantly increased after GNE-987 treatment. **F** Annexin V and PI-labeled cell apoptosis of MV4–11 cell line analyzed by flow cytometry after DMSO or GNE-987 treatment for 24 h. The apoptotic rates of MV4–11 cell line were significantly increased after GNE-987 treatment. **G** GNE-987 evicts BET protein expression in AML cells. Western blotting analysis showed that GNE-987 induced BET proteins degradation in NB4, Kasumi-1, HL-60, and MV4–11 cell lines. PARP was increased in NB4, Kasumi-1, HL-60, and MV4–11 cell lines with GNE-987 concentration-dependent manner

To evaluate the dependence of AML cell growth on transcriptional activation, we next examined the effect of the BRD4 inhibitor GNE-987 on AML cells. We chose four AML cell lines, NB4, Kasuma-1, HL-60, and MV4–11. In this analysis, all the four AML cell lines showed high sensitivity to GNE-987, with 50% inhibitory concentrations of less than 50 nM (Fig. 3A). Cell cycle analysis indicated that GNE-987 treatment led to cell-cycle arrest in G1 phase (Fig. 3B). Of note, apoptosis rates of AML cells were significantly increased after GNE-987 treatment (Fig. 3C, D, E, F). Western blotting analysis also showed PARP cleavage after GNE-987 treatment in the four AML cell lines (Fig. 3G), indicating that GNE-987 induced apoptotic cell death. These results indicated that GNE-987 treatment predominantly inhibits the growth of AML cells.

### GNE-987 treatment inhibits the expression of super-enhancer-associated genes in AML cells

**Fig. 4:**
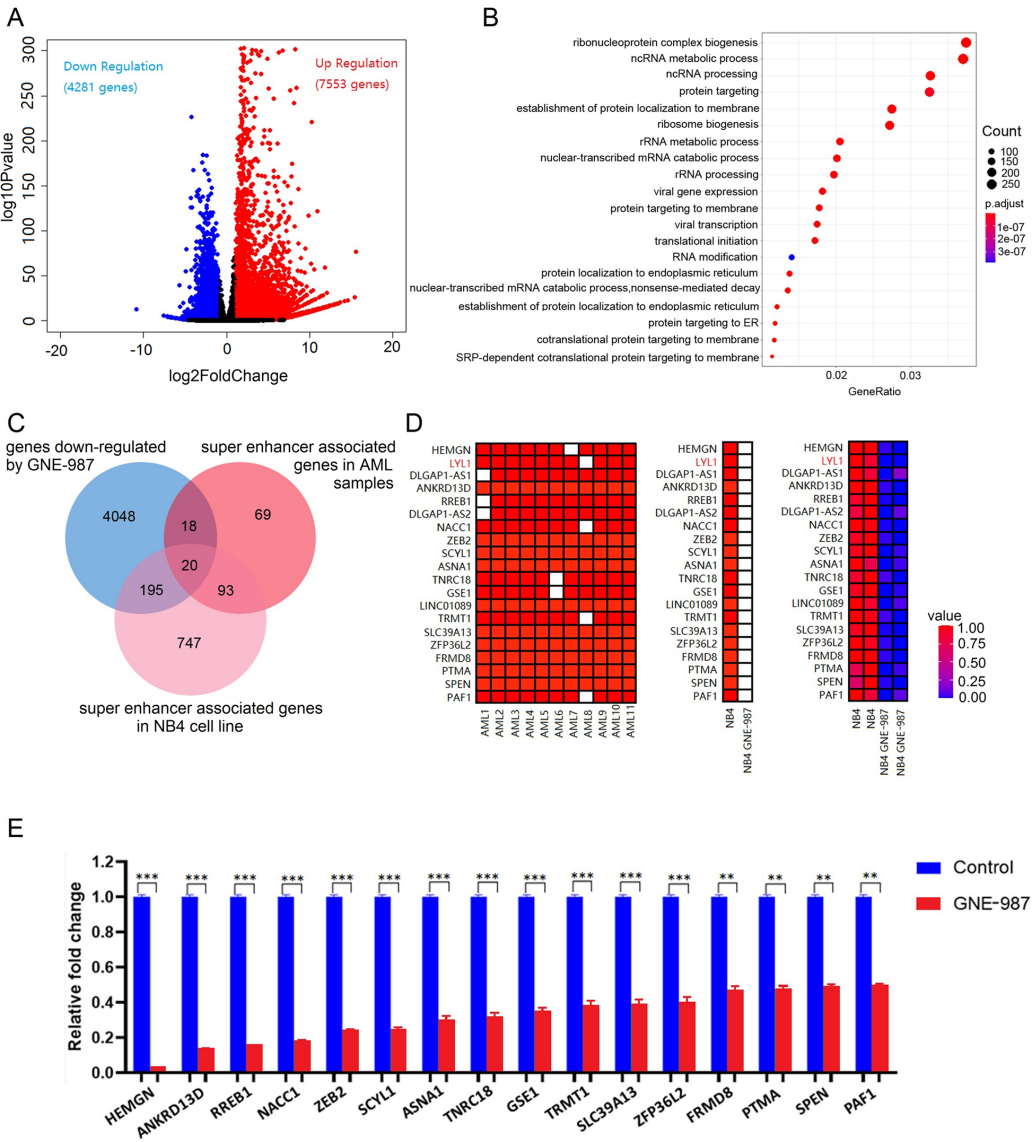
**A** Volcano Plot of RNA-seq results for NB4 cell line in either the absence or presence of GNE-987 (25 nM, 24 h). **B** Gene oncology enrichment analysis results of all DEGs. **C** Venn diagram of genes associated with super-enhancers and sensitive to GNE-987 in AML cells. **D** Super-enhancer status at 20 gene loci in 11 AML samples and NB4 cells (left and middle). Super-enhancers are shown in red. A heatmap represents the relative expression levels of the 20 genes after GNE-987 treatment in NB4 cells (right). **E** qRT-PCR results of 16 super-enhancer-associated genes in NB4 cell line treated with GNE-987

We next carried out RNAseq to obtain gene expression profiles after GNE-987 treatment in NB4 cells. A total of 11,834 genes were identified to be differentially expressed between the control group and the GNE-987-treated group (Fig. 4A, Supplementary Table 10). Through gene ontology enrichment analysis, these 11,834 genes were found to be enriched in ribonucleoprotein complex biogenesis (Fig. 4B). Notably, the expression levels of the super-enhancer-associated genes *HEMGN*, *LYL1*, *ANKRD13D*, *RREB1*, *NACC1*, *ZEB2*, *SCYL1*, *ASNA1*, *TNRC18*, *GSE1*, *TRMT1*, *SLC39A13*, *ZFP36L2*, *FRMD8*, *PTMA*, *SPEN* and *PAF1* were significantly downregulated after GNE-987 treatment based on the RNAseq restuls (Fig. 4C,D). qRT-PCR validation further showed that these genes were downregulated in NB4 cells treated with GNE-987 (Fig. 4E). These findings suggest that GNE-987 efficiently downregulates the expression of super-enhancer-associated genes in AML cells.

### Selection of candidate cancer genes in AML by super-enhancer and gene expression profile

Our ChIP-seq results suggested that many genes involved in AML pathogenesis are correlated with super-enhancers. The expression of these genes was significantly inhibited by GNE-987 treatment. Therefore, we next combined super-enhancer profiling with gene expression profiling to identify critical cancer genes implicated in AML pathogenesis. We focused on the 200 genes that were associated with super-enhancers in ≧ 10 AML samples (Fig. 4C; Supplemental Table 7). Additionally, we performed H3K27ac ChIP-Seq after GNE-987 treatment in NB4 cells, and performed filtering to identify genes that also harbor super-enhancers in NB4 cells (Fig. 4C, Supplementary Table 11). We next performed filtering to identify genes that were also significantly downregulated after GNE-987 treatment (*P* value < 0.05, log2 fold change <− 1) in NB4 cells (Fig. 4C). Filtering according to the above stringent criteria narrowed down the list of candidates to 20 genes (Fig. 4C, D).

### LYL1 is required for AML cell growth and survival

**Fig. 5:**
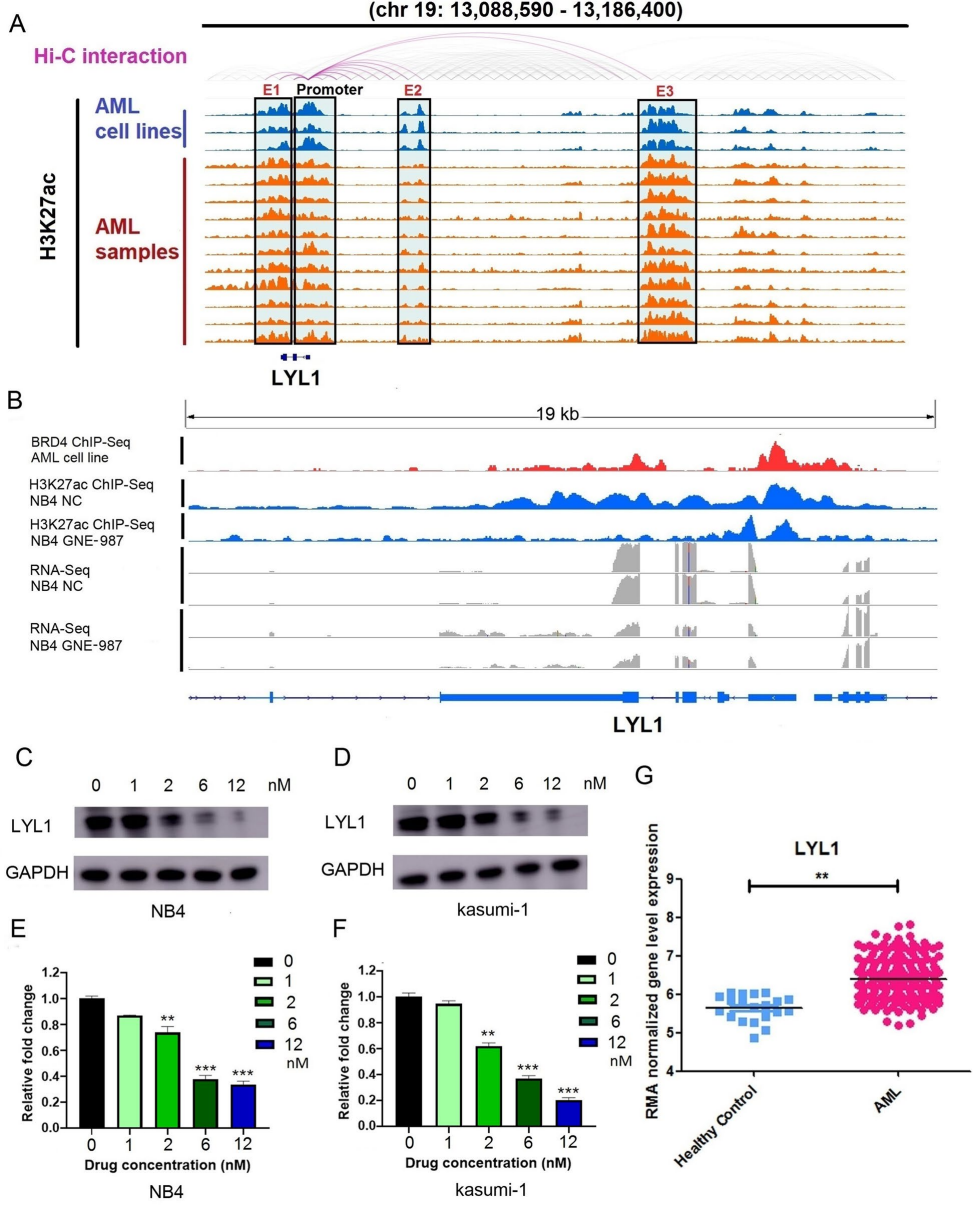
**A** The Hi-C data analysis of super-enhancer regions and promoter of *LYL1* in THP-1 cell line (track 1) represents interaction at the *LYL1* gene loci, and the ChIP-seq gene tracks (track 2–15) represent the H3K27ac signal in AML cell lines (NB4, MV4–11, and THP1) and 11 AML samples at the *LYL1* gene loci. The super-enhancers are shown as green boxes. **B** The public BRD4 ChIP-Seq data of AML cell line MV4–11 (GSE101821) showed that the gene region of *LYL1* had coincident signals (track 1). The ChIP-seq gene tracks represent the H3K27ac signal in NB4 cells (track 2) and NB4 cells treated with GNE-987 (25 nM, 24 h) (track 3) at the *LYL1* gene loci. The reads signal of RNA-Seq in NB4 cells (track 4–5) and NB4 cells treated with GNE-987 (25 nM, 24 h) (track 6–7) at the *LYL1* gene loci. **C** Western blotting showed that LYL1 level was significantly decreased in NB4 cells treated with GNE-987 (0, 1, 2, 6, 12 nM, 24 h). **D** Western blotting showed that LYL1 level was significantly decreased in Kasumi-1 cells treated with GNE-987 (0, 1, 2, 6, 12 nM, 24 h). **E** qPCR showed that *LYL1* was significantly downregulated in NB4 cells treated with GNE-987 (0, 1, 2, 6, 12 nM, 24 h). **F** qPCR showed that *LYL1* was significantly downregulated in Kasumi-1 cells treated with GNE-987 (0, 1, 2, 6, 12 nM, 24 h). **G** expression pattern of *LYL1* between AML patients and healthy controls in public transcriptomic dataset (GSE114868)

**Fig. 6:**
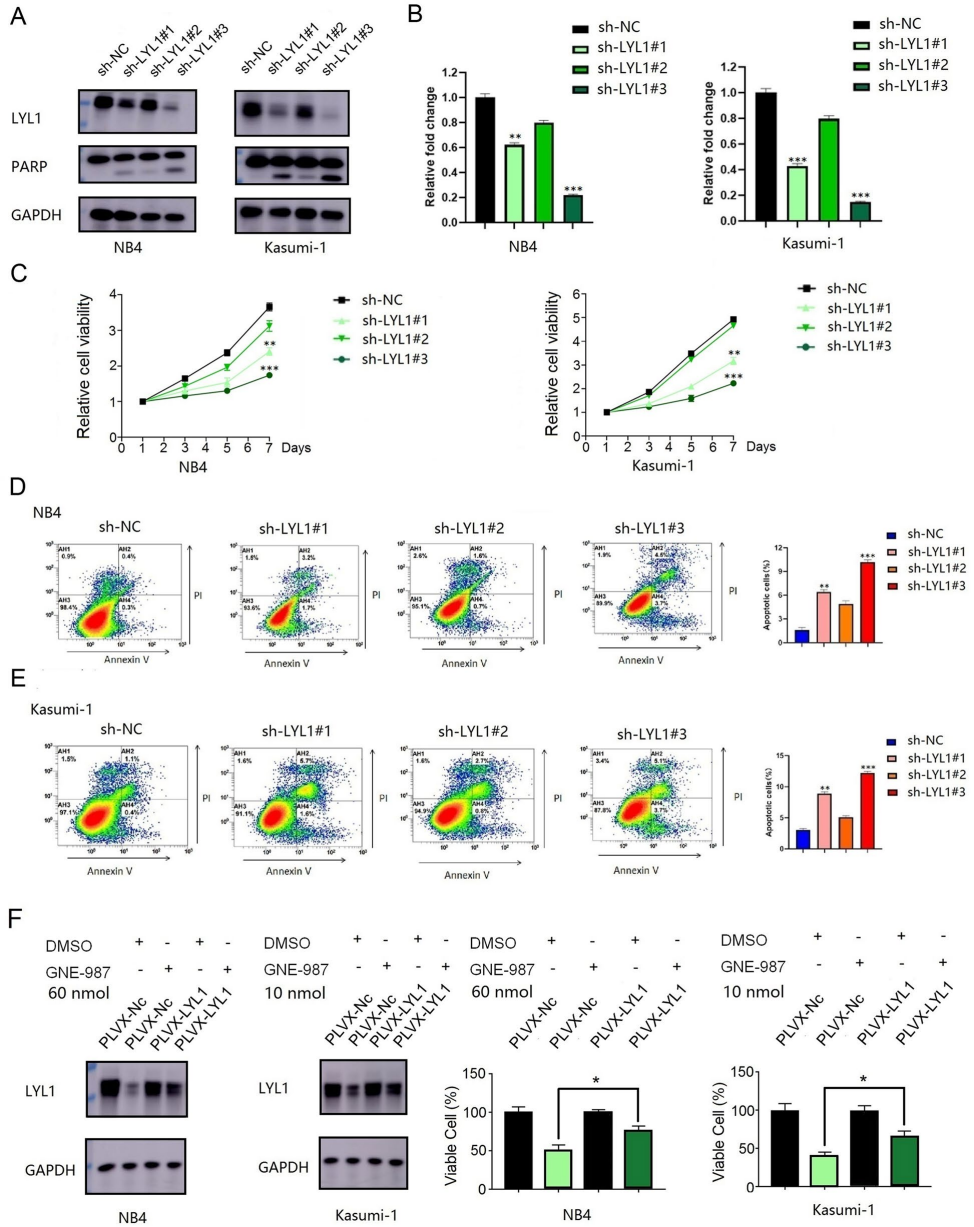
**A** Knockdown efficiency of *LYL1* was evaluated in NB4 cell line and Kasumi-1 cell line by western blotting. **B** Knockdown efficiency of *LYL1* was evaluated in NB4 cell line and Kasumi-1 cell line by qPCR. **C** Knockdown of *LYL1* significantly inhibited the proliferation rates of NB4 cells and Kasumi-1 cells. **D** Flow cytometry showed that knockdown of *LYL1* increased the apoptotic rates of NB4 cells. **E** Flow cytometry showed that knockdown of *LYL1* increased the apoptotic rates of Kasumi-1 cells. **F** Cell viability experiment further showed that cell growth was not significantly influenced by GNE-987 (treated for 24 h) in *LYL1* over-expressed AML cells, compared to *LYL1* non-overexpressed AML cells

Among those 20 genes, *LYL1* was associated with super-enhancers in 10 AML samples (Figs. 4D and 5A). The results of Hi-C data also represent interaction between super-enhancer and *LYL1* in THP-1 cell line. Super-enhancers associated with *LYL1* were common to all three hematological diseases (AML, T-ALL, and B-ALL) (Supplementary Fig. 4). The public BRD4 ChIP-Seq data for the AML cell line MV4–11 (GSE101821) showed that the gene region of *LYL1* had strong signals (Fig. 5B, track 1), and BRD4 was found to function cooperatively with CEBPE and RUNX1 (Supplementary Fig. 5), suggesting a potential role for BRD4 in the transcriptional regulation of *LYL1* in AML. *LYL1* also harbored super-enhancer in NB4 cells, and the H3K27ac signal was significantly decreased in the *LYL1* gene region in NB4 cells treated with GNE-987 (Figs. 4D and 5B (tracks 2–3)). In fact, *LYL1* expression was significantly downregulated by GNE-987 treatment in both the NB4 and Kasumi-1 cell lines (Fig. 5B (tracks 4–7), 5C, 5D, 5E, 5F). We compared the expression pattern of *LYL1* between AML cases and healthy controls based on a public transcriptomic dataset (GSE114868) [33] and found that *LYL1* was significantly overexpressed in AML samples (Fig. 5G). Knockdown of *LYL1* demonstrated that loss of LYL1 significantly inhibited the growth of NB4 and Kasumi-1 cell lines (Fig. 6A, B, C). Consistently with this finding, the apoptosis rates of NB4 and Kasumi-1 cells were significantly increased after *LYL1* knockdown (Fig. 6D, E). Cell viability experiment further showed that cell growth was not significantly influenced by GNE-987 (treated for 24 h) in *LYL1* over-expressed AML cells, compared to *LYL1* non-overexpressed AML cells (Fig. 6F), suggesting that the effect of GNE-987 on AML cell growth depends in part on *LYL1*.

**Fig. 7:**
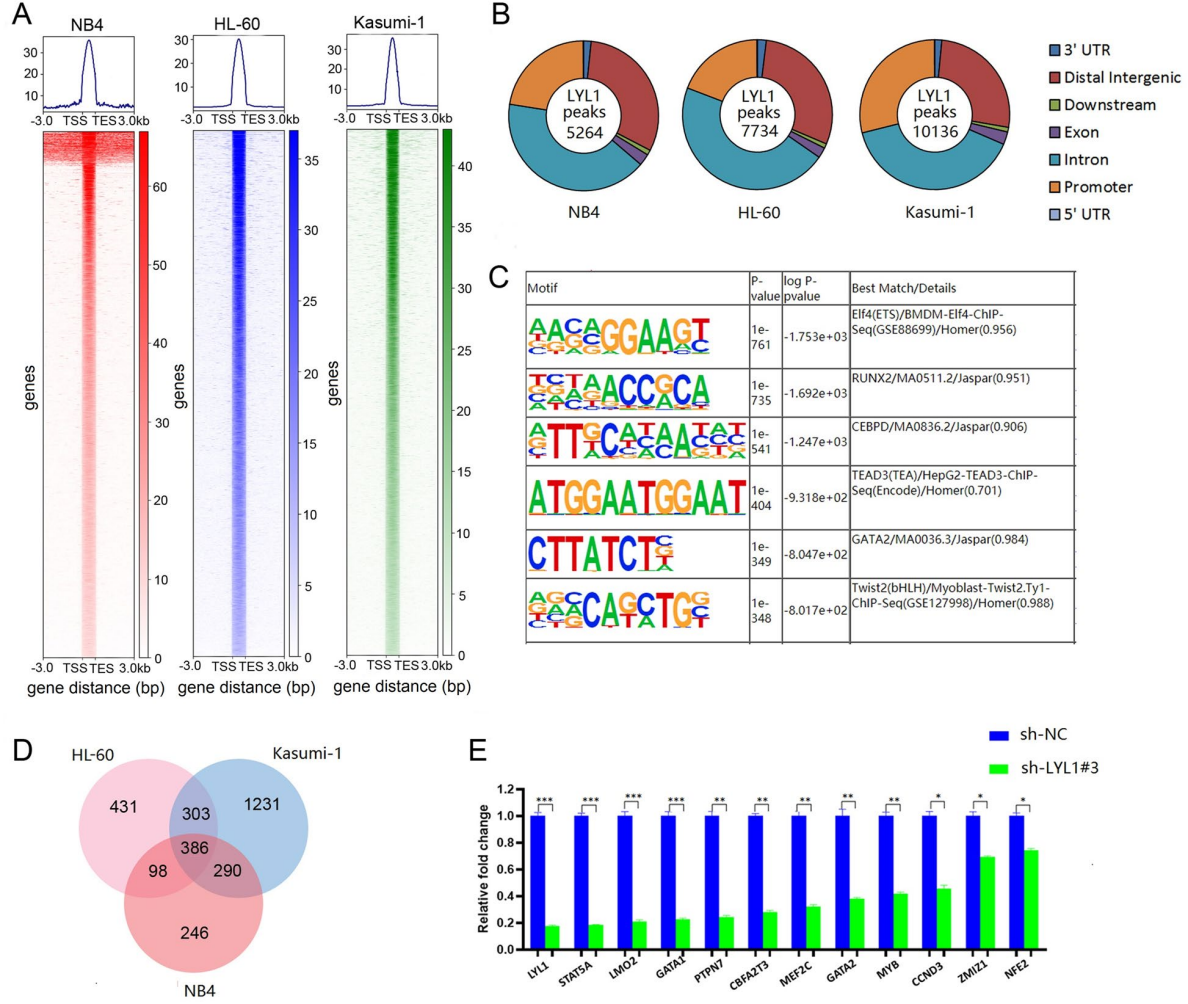
**A** Heatmaps generated from ChIP-Seq data analyses showed the occupancy of LYL1 in NB4, HL-60, and Kasumi-1 cell lines. **B** distribution of LYL1 binding to genomic regions in the human AML cell line NB4, HL-60, and Kasumi-1 as assessed by ChIP-Seq. Peak annotation of LYL1 is summarized in a pie chart format. **C** Motif enrichment analysis of the 5264 specific LYL1 peaks in NB4 cells. **D** Venn diagram of common genes bound by LYL1 at its promoter regions in AML cell line NB4, HL-60, and Kasumi-1. **E** qRT-PCR results of 11 genes in NB4 cell line when silencing *LYL1*

We next assessed the binding pattern of LYL1 across genomic regions in three public ChIP-Seq datasets (for the NB4, HL-60 and Kasumi-1 cell line respectively, GSE63484) [31] and found that LYL1 functions cooperatively with Elf4, RUNX2, CEBPD, TEAD3, GATA2, and Twist2 (Fig. 7A, B, C). Importantly, LYL1 was found to bind to the promoter region of 386 genes in all three AML cell lines (NB4, HL-60, and Kasumi-1) (Fig. 7D, Supplementary Table 12). qRT-PCR validation further showed that these genes were significantly downregulated in NB4 cells in response to *LYL1* silencing (Fig. 7E). Together, these results suggest that *LYL1* is required for the growth and survival of human AML cells.

## Discussion

AML is an aggressive neoplasm and its prognosis is poor [8]. AML is complex, and exploring its pathogenic mechanisms will help improve the current state of AML treatment [5–8]. Multiple hub genes have been identified in AML. RUNX1 dysfunction is a major pathogenic mechanism of AML [9]. MPO is a marker for AML diagnosis and prognosis [11]. CDK6 is another key molecule in AML development [12].

Super-enhancers are attracting the attention of many researchers at present. Super-enhancers recruit a particularly large number of transcription factors/cofactors and induce the transcription of many target genes, compared with typical enhancers. Super-enhancers have been reported to be frequently associated with cancer genes [28, 34–36]. Super-enhancer promotes the growth and survival of t(4;14)-positive multiple myeloma [16]. Super-enhancer was found to activate the Wnt/beta-catenin pathway and promotes the proliferation of liver cancer cells [17]. Oncogenic super-enhancers were also identified in colorectal cancer through genome-wide profiling [18]. In addition, super-enhancer was reported to play a role in glioma progression [19]. Super-enhancer was also found to be involved in squamous cell carcinoma [20]. Furthermore, super-enhancer is known to play a role in triple-negative breast cancer [21]. In addition, super-enhancer has been reported to be abnormally activated and result in CHPT1 overexpression, which leads to enzalutamide resistance in castration-resistant prostate cancer [22]. To date, the biological significance of super-enhancers in AML remains unclear, and it is useful to identify critical super-enhancers and associated genes that are required for the development of AML.

In our study, we found super-enhancers at 200 gene loci in ≧ 10 AML samples. These genes are required for AML progression. Strikingly, we identified super-enhancers at the *LYL1* gene locus in 10 AML samples. We also found that GNE-987 treatment downregulates the expression of super-enhancer-associated genes in AML cells, including the expression of *LYL1*. Further functional analysis indicated that *LYL1* is required for AML cell growth and survival. These results elucidated a crucial role of *LYL1* in promoting AML progression. The ChIP-Seq results in AML cell lines differed from those in AML patient samples in this study, and further ChIP-Seq analysis of AML cell lines is necessary to confirm this discrepancy.

*LYL1* was identified to function in clear cell renal cell carcinoma [37]. It was reported to have strong association with immune infiltrations in clear cell renal cell carcinoma. Copy number amplification of *LYL1* was reported in gliosarcoma based on a comparison at the molecular level between gliosarcoma patients and glioblastoma patients [38]. *LYL1* expression also showed differences between osteosarcoma and control samples, and the expression of *LYL1* was significantly decreased in osteosarcoma cell lines compared to normal cells [39]. *LYL1* gene amplification was also determined to be involved in the development of uterine corpus endometrial carcinoma, and *LYL1* gene amplification was reported to be a risk factor for poor prognosis in patients with uterine corpus endometrial carcinoma according to a study of 370 patients with uterine corpus endometrial carcinoma [40]. *LYL1* is able to maintain primitive erythropoiesis, and it was reported to bind to a subset of stem cell leukemia (SCL) targets according to ChIP-seq analysis in a human erythroleukemia cell line [41]. *LYL1* functions in platelet production in mice [42]. It regulates GATA1 expression and functions cooperatively with SCL in platelet production. It has been reported that the expression of *LYL1* is higher in AML than in normal bone marrow, and *LYL1* was found to be overexpressed in myelodysplastic syndrome compared with normal bone marrow [43]. LYL1 is a member of a heptad of transcription factors that play roles in human CD34+ haematopoietic stem and progenitor cells (HSPCs), and LYL1 has prognostic significance in AML [44]. In this study, *LYL1* was found to be associated with super-enhancers in 10 AML samples, suggesting that aberrant expression of *LYL1* triggered by super-enhancers probably plays a role in AML development. It has also been reported that transcriptional regulation can be both BRD4-dependent and BRD4-independent [45]; therefore the expression of *LYL1* might be regulated in both BRD4-dependent and BRD4–independent manners. To our knowledge, there is no reported enhancer regulation of *LYL1* to date, and whether the expression of *LYL1* is also regulated by the BRD4-independent enhancer needs to be explored in future studies.

## Conclusion

In summary, we identified 200 common super-enhancer-associated genes in AML samples, and a series of those genes are cancer genes. We also found that GNE-987 treatment downregulates the expression of super-enhancer-associated genes in AML cells, including the expression of *LYL1*. Further functional analysis indicated that *LYL1* is required for AML cell growth and survival. These findings promote the understanding of AML pathophysiology and elucidate an important role of *LYL1* in AML progression.

## Supplementary Information

**Additional file 1: Supplementary Table 1.** Treatment and outcome of 11 pediatric AML patients included in ChIP-seq analysis.

**Additional file 2: Supplementary Table 2.** shRNAs used to knockdown *LYL1* and PLVX-LYL1 used to over-express *LYL1*.

**Additional file 3: Supplementary Table 3.** Primers used for qRT-PCR analyses.

**Additional file 4: Supplementary Table 4.** Super-enhancers identified in each of the 11 AML samples.

**Additional file 5: Supplementary Table 5.** Super-enhancers identified in each of the 7 T-ALL samples.

**Additional file 6: Supplementary Table 6.** Super-enhancers identified in each of the 8 B-ALL samples.

**Additional file 7: Supplementary Table 7.** 200 hundred genes which harbor super-enhancers in ≧ 10 AML samples.

**Additional file 8: Supplementary Table 8.** Gene ontology enrichment analysis results of the 200 genes which were commonly associated with super-enhancers in ≧10 AML samples.

**Additional file 9: Supplementary Table 9.** FPKM values from RNAseq results of 9 AML samples.

**Additional file 10: Supplementary Table 10.** 11,834 genes identified to be differentially expressed between the control and GNE-987-treated NB4 cells.

**Additional file 11: Supplementary Table 11.** Super-enhancer-associated genes in NB4 cells and in NB4 cells treated with GNE-987.

**Additional file 12: Supplementary Table 12.** Genes bound by LYL1 at its promoter regions in AML cell line NB4, HL-60, and Kasumi-1.

**Additional file 13: Supplementary Figure 1.** Compared to T-ALL or B-ALL, super-enhancers associated with *MPO* are observed to be AML specific.

**Additional file 14: Supplementary Figure 2.** Super-enhancers associated with *ZFP36L2* are common to all three hematological diseases (AML, T-ALL, and B-ALL).

**Additional file 15: Supplementary Figure 3.** Compared to T-ALL, super-enhancers associated with *SPI1* are found to be AML and B-ALL specific.

**Additional file 16: Supplementary Figure 4.** Super-enhancers associated with *LYL1* are common to all three hematological diseases (AML, T-ALL, and B-ALL).

**Additional file 17: Supplementary Figure 5.** The public BRD4 ChIP-Seq data of AML cell line MV4–11 (GSE101821) showed that BRD4 functions with CEBPE and RUNX1.

## Data Availability

The data used and/or analyzed during the current study are available from the corresponding author on reasonable request (GSE188605, GSE188750, GSE188891, GSE190775).

## Abbreviations

AML: Acute myeloid leukemia
ChIP: Chromatin immunoprecipitation
LYL1: LYL1 basic helix-loop-helix family member
BM: Bone marrow
SE: Super-enhancer
TE: Typical enhancer
