# Supplementary material for: Super-enhancer profiling identifies novel critical and targetable cancer survival gene LYL1 in pediatric acute myeloid leukemia
Source: J Exp Clin Cancer Res. 2022 Jul 16;41:225. doi: 10.1186/s13046-022-02428-9 (PMC9288051; doi:10.1186/s13046-022-02428-9)
Supplement: Supplementary file 2 — Additional file 2: Supplementary Table 2. shRNAs used to knockdown LYL1 and PLVX-LYL1 used to over-express LYL1. [file 13046_2022_2428_MOESM2_ESM.docx]

**Supplementary Table 2.** shRNAs used to knockdown *LYL1* and PLVX-LYL1 used to over-express *LYL1*

| Name | Sequence |
| --- | --- |
| Homo-LYL1-sh1 | CCGGAGAAGGCAGAGATGGTGTGTGCTCGAGCACACACCATCTCTGCCTTCTTTTTTTGAATT |
| Homo-LYL1-sh2 | CCGGCACTTTGGCCCTGCACTACCACTCGAGTGGTAGTGCAGGGCCAAAGTGTTTTTTGAATT |
| Homo-LYL1-sh3 | CCGGCTTCCTCAACAGTGTCTACATCTCGAGATGTAGACACTGTTGAGGAAGTTTTTGAATT |
| PLVX-LYL1 | GAATTCGCCACCATGTGCCCGCCTCAGGCACAGGCAGAGGTGGGCCCCACCATGACTGAGAAGGCAGAGATGGTGTGTGCCCCCAGCCCAGCGCCTGCCCCACCCCCTAAGCCTGCCTCGCCTGGGCCCCCGCAGGTGGAGGAGGTGGGCCACCGAGGAGGCTCCTCGCCCCCCAGGCTGCCACCTGGTGTACCAGTGATCAGCCTGGGCCACAGCAGGCCCCCAGGGGTAGCCATGCCCACCACAGAGCTGGGCACTCTGCGGCCCCCGCTGCTGCAACTCTCCACCCTGGGAACTGCCCCGCCCACTTTGGCCCTGCACTACCACCCTCACCCCTTCCTCAACAGTGTCTACATTGGGCCAGCAGGACCTTTTAGCATCTTCCCTAGCAGCCGGTTGAAGCGGAGACCAAGCCACTGTGAGCTGGACCTGGCTGAGGGGCACCAGCCCCAGAAGGTGGCCCGGCGCGTGTTCACCAACAGCCGGGAGCGCTGGCGGCAGCAGAACGTTAACGGCGCCTTCGCCGAGCTGAGGAAGCTGCTGCCGACGCACCCGCCCGACCGGAAGCTGAGCAAGAACGAGGTGCTCCGCCTAGCCATGAAGTACATCGGCTTCCTGGTGCGGCTGCTGCGCGACCAAGCCGCAGCTCTGGCCGCAGGCCCCACCCCTCCCGGGCCTCGCAAACGGCCGGTGCACCGGGTCCCAGACGACGGCGCCCGCCGGGGATCCGGACGCAGGGCCGAGGCGGCAGCGCGCTCGCAGCCCGCGCCCCCGGCCGACCCCGACGGCAGCCCCGGTGGAGCGGCCCGGCCCATCAAGATGGAGCAAACCGCTTTGAGCCCAGAGGTGCGGGACTACAAAGACCATGACGGTGATTATAAAGATCATGATATCGATTACAAGGATGACGATGACAAGTGAGCGGCCGC |
