# Supplementary material for: Super-enhancer profiling identifies novel critical and targetable cancer survival gene LYL1 in pediatric acute myeloid leukemia
Source: J Exp Clin Cancer Res. 2022 Jul 16;41:225. doi: 10.1186/s13046-022-02428-9 (PMC9288051; doi:10.1186/s13046-022-02428-9)
Supplement: Supplementary file 3 — Additional file 3: Supplementary Table 3. Primers used for qRT-PCR analyses. [file 13046_2022_2428_MOESM3_ESM.docx]

**Supplementary Table 3.** Primers used for qRT-PCR analyses

| Name | Sequence (5’->3’) |
| --- | --- |
| LYL1 Forward | ACAGTGTCTACATTGGGCCAG |
| LYL1 Reverse | GGCTGCTAGGGAAGATGCT |
| STAT5A Forward | CAGTGGTTTGACGGGGTGAT |
| STAT5A Reverse | GTCGTGGGCCTGTTGCTTAT |
| LMO2 Forward | GGCCATCGAAAGGAAGAGCC |
| LMO2 Reverse | GGCCCAGTTTGTAGTAGAGGC |
| GATA1 Forward | CTGTCCCCAATAGTGCTTATGG |
| GATA1 Reverse | GAATAGGCTGCTGAATTGAGGG |
| CBFA2T3 Forward | CACTCACCAACAGCCATCAAT |
| CBFA2T3 Reverse | CGTCAATGTCGAGTTCACCAG |
| MEF2C Forward | GAACGTAACAGACAGGTGACAT |
| MEF2C Reverse | CGGCTCGTTGTACTCCGTG |
| GATA2 Forward | GCAACCCCTACTATGCCAACC |
| GATA2 Reverse | CAGTGGCGTCTTGGAGAAG |
| MYB Forward | ATCTCCCGAATCGAACAGATGT |
| MYB Reverse | TGCTTGGCAATAACAGACCAAC |
| CCND3 Forward | TACCCGCCATCCATGATCG |
| CCND3 Reverse | AGGCAGTCCACTTCAGTGC |
| ZMIZ1 Forward | TGTTTGACGGTGGTCAGTCG |
| ZMIZ1 Reverse | CTTGTCTCGGTTTGCAGCAC |
| NFE2 Forward | CGGCGCAGCGAATATGTAGA |
| NFE2 Reverse | CCGACGTTCATCCCGACTC |
| PTPN7 Forward | GGGAGGTCACCCTACACTTTC |
| PTPN7 Reverse | TGGTCTTGTATCGGTCCTTGG |
| HEMGN Forward | AGCTGAAGTGCATGAAAAGGAA |
| HEMGN Reverse | ACTTTGGTTATGCTCCCAGGT |
| ANKRD13D Forward | CAGCACGACATTGAACAGGAG |
| ANKRD13D Reverse | GAAGGAGCACTCTCACAGACT |
| RREB1 Forward | AGGTTCAGACCTATCTTCCATCA |
| RREB1 Reverse | CTGCCAATCCGATTTGGTCCT |
| NACC1 Forward | CTGGCTCCTACCACAATGAGG |
| NACC1 Reverse | TGGCCGACGTTCATCATGC |
| ZEB2 Forward | CAAGAGGCGCAAACAAGCC |
| ZEB2 Reverse | GGTTGGCAATACCGTCATCC |
| SCYL1 Forward | TGACCCCGTTGGGAATATACC |
| SCYL1 Reverse | GAGGGCTTTCACGATCTGGTG |
| ASNA1 Forward | GCCTACCAAGGTCAAAGGCTA |
| ASNA1 Reverse | CGTGTCAAATACCACCACCG |
| TNRC18 Forward | CCTCCGCTTTTCGGCAAGA |
| TNRC18 Reverse | GCACATGGTCAGTACCGACG |
| TRMT1 Forward | AGGATCGTCTCTGTGGCTAAG |
| TRMT1 Reverse | TGGACGTTCTTCTCCGTAGGG |
| SLC39A13 Forward | TCAGCGGCTACCTCAACCT |
| SLC39A13 Reverse | AGGAGCCCGATCTTCTTGCT |
| ZFP36L2 Forward | GAGAACAAATTCCGGGACCG |
| ZFP36L2 Reverse | GCGTGGAGTTGATCTGGGAG |
| FRMD8 Forward | CGGGGCCAGAGTCTCTTTG |
| FRMD8 Reverse | TCGTGGCACTTGAGGAGATAG |
| PTMA Forward | TCAGACGCAGCCGTAGACA |
| PTMA Reverse | GCATTCCCGTTAGCAGGGG |
| SPEN Forward | CGAGCATTTCAAACGATATGGC |
| SPEN Reverse | CCATTTTGTTGACCGAGTTGTG |
| PAF1 Forward | ACCCCTTCGACCAGAACAG |
| PAF1 Reverse | CGGTAGGTGTCAGGATTGATGAG |
| GSE1 Forward | GAGCGCCTATTGGTGGAGC |
| GSE1 Reverse | CTGGAGGTTGAGTGGTTGGAA |
| GAPDH Forward | ACAACTTTGGTATCGTGGAAGG |
| GAPDH Reverse | GCCATCACGCCACAGTTTC |
