# Supplementary figures and images for: Super-enhancer profiling identifies novel critical and targetable cancer survival gene LYL1 in pediatric acute myeloid leukemia
Source: J Exp Clin Cancer Res. 2022 Jul 16;41:225. doi: 10.1186/s13046-022-02428-9 (PMC9288051; doi:10.1186/s13046-022-02428-9)

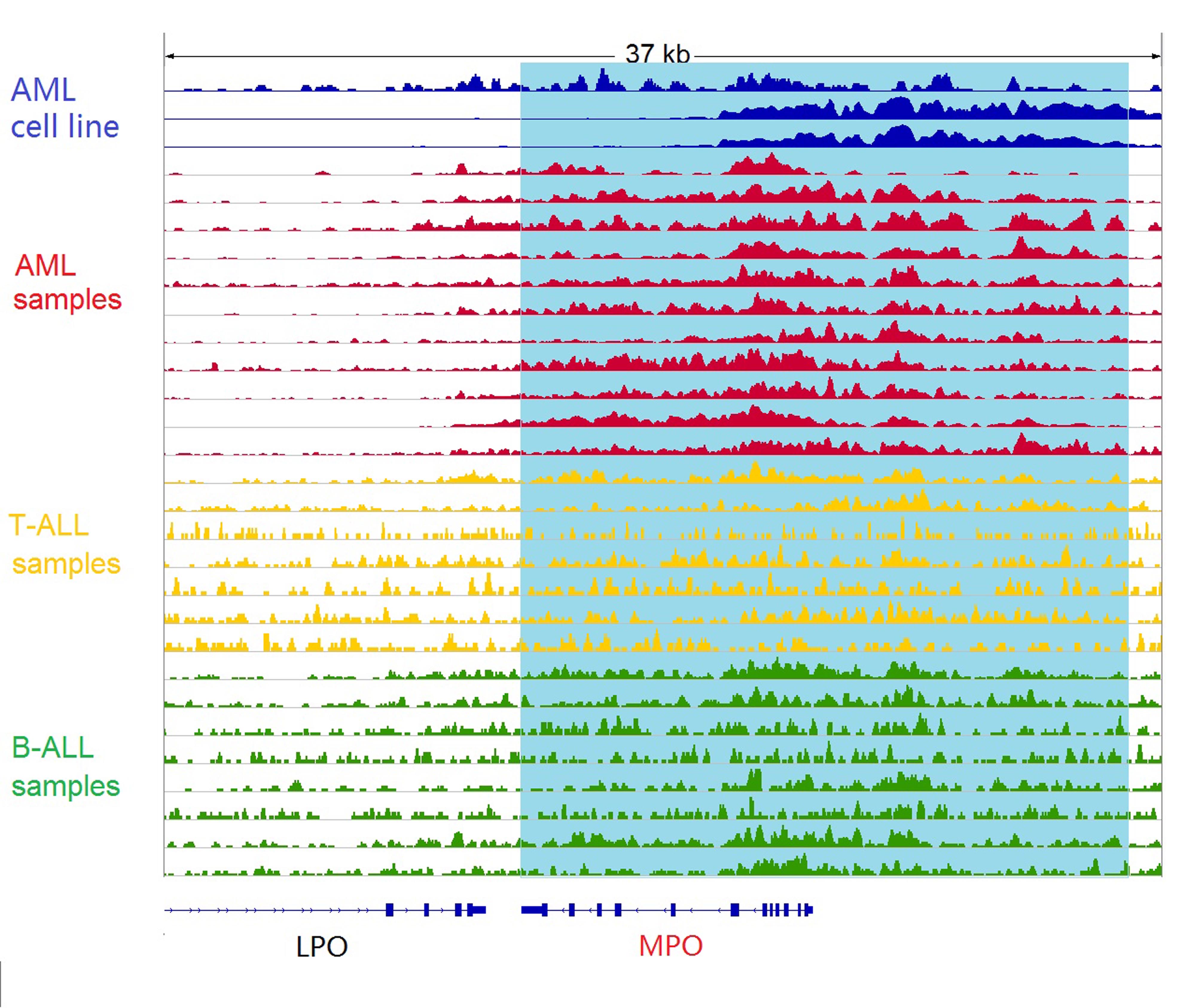

Supplement: Supplementary file 13 — Additional file 13: Supplementary Figure 1. Compared to T-ALL or B-ALL, super-enhancers associated with MPO are observed to be AML specific. [file 13046_2022_2428_MOESM13_ESM.jpg]

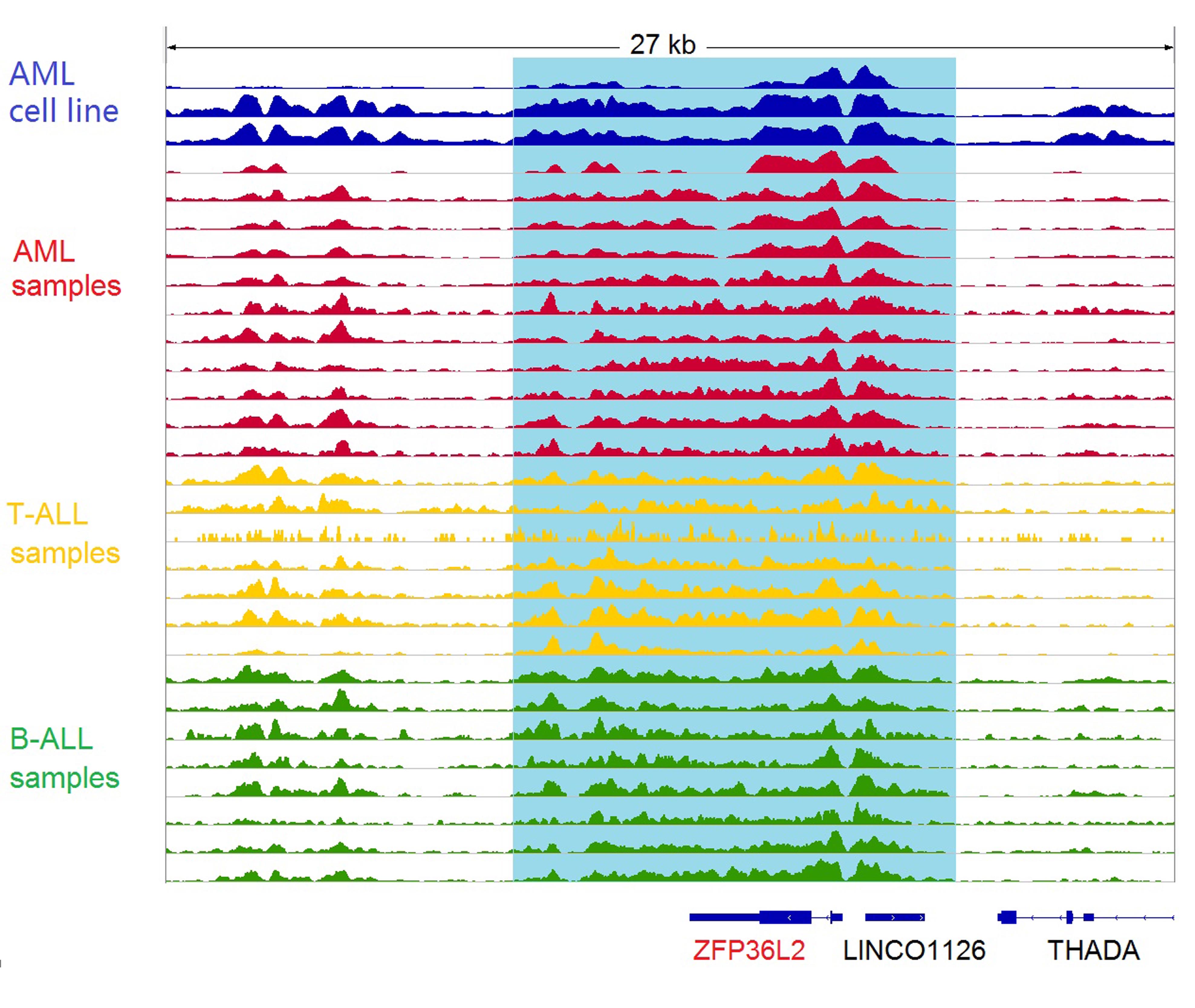

Supplement: Supplementary file 14 — Additional file 14: Supplementary Figure 2. Super-enhancers associated with ZFP36L2 are common to all three hematological diseases (AML, T-ALL, and B-ALL). [file 13046_2022_2428_MOESM14_ESM.jpg]

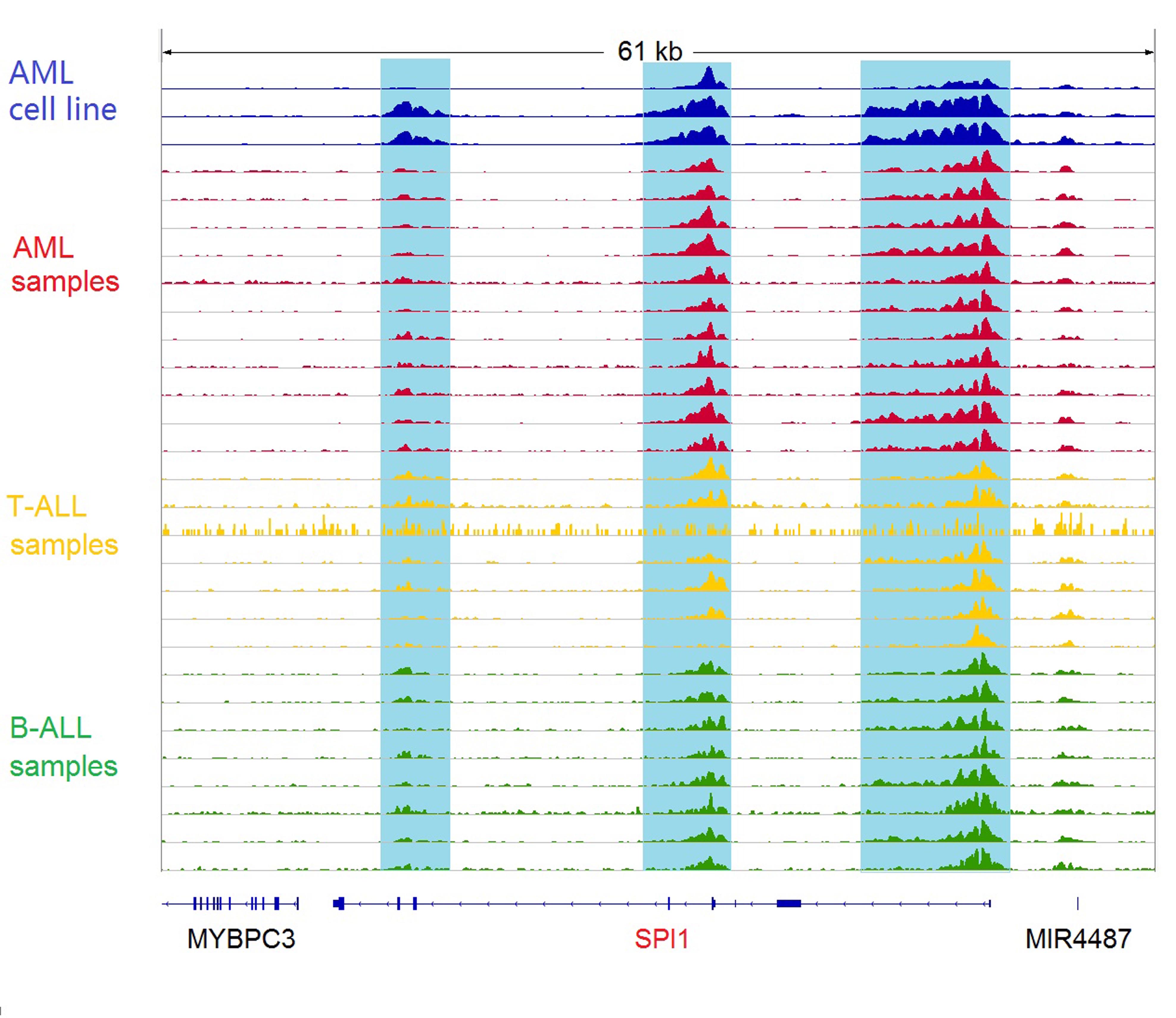

Supplement: Supplementary file 15 — Additional file 15: Supplementary Figure 3. Compared to T-ALL, super-enhancers associated with SPI1 are found to be AML and B-ALL specific. [file 13046_2022_2428_MOESM15_ESM.jpg]

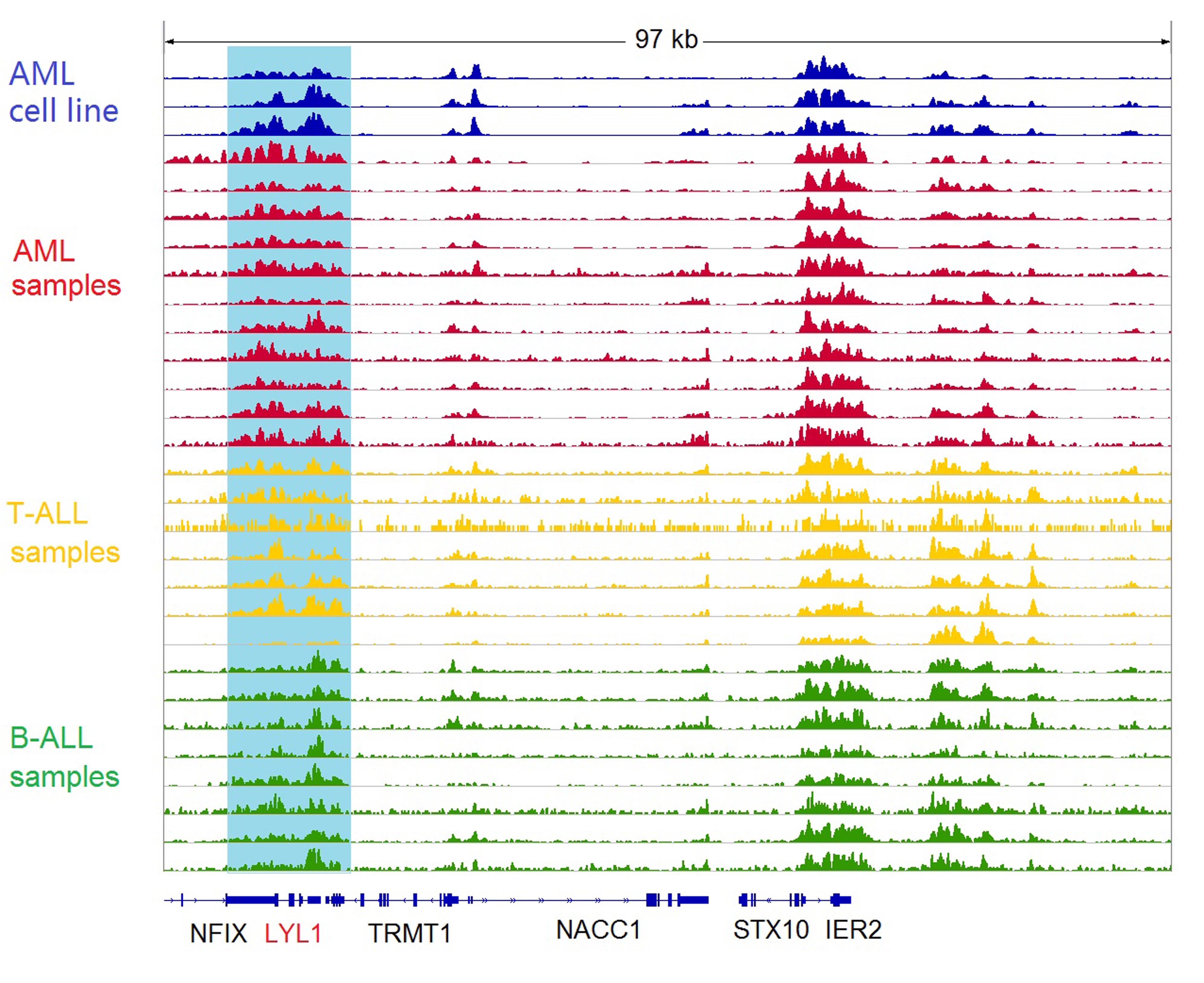

Supplement: Supplementary file 16 — Additional file 16: Supplementary Figure 4. Super-enhancers associated with LYL1 are common to all three hematological diseases (AML, T-ALL, and B-ALL). [file 13046_2022_2428_MOESM16_ESM.jpg]

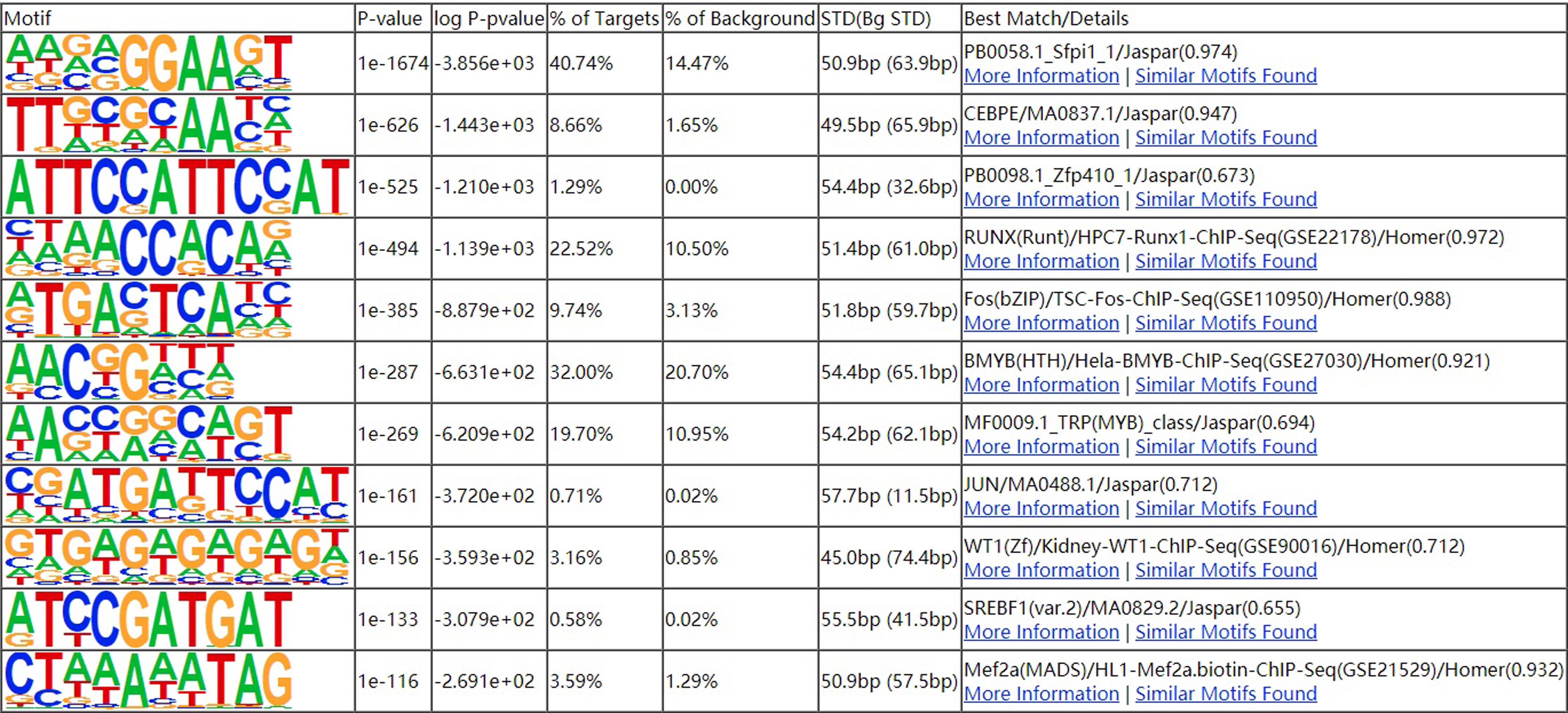

Supplement: Supplementary file 17 — Additional file 17: Supplementary Figure 5. The public BRD4 ChIP-Seq data of AML cell line MV4–11 (GSE101821) showed that BRD4 functions with CEBPE and RUNX1. [file 13046_2022_2428_MOESM17_ESM.jpg]
